# AUTOLOGOUS SERUM SKIN TEST

**DOI:** 10.4103/0019-5154.49000

**Published:** 2009

**Authors:** Sudip Kumar Ghosh, Sanjay Ghosh

**Affiliations:** *From the Department of Dermatology, Venereology, and Leprosy, R.G. Kar Medical College, kolkata, India*; 1 *From the Department of Institute of Allergic and Immunologic Skin Disease, Kolkata, India*

## Introduction

About one third of the patients of chronic urticaria have circulating functional histamine-releasing autoantibodies against high-affinity IgE receptor, or less commonly against IgE.[1] Autoantibodies in patients’ serum can be detected by serum-induced histamine release from the basophils of healthy donors by ELISA[2] or Western blot assay.[3] But neither Western blot nor ELISA can differentiate between functional histamine-releasing autoantibody and nonfunctional autoantibody. Moreover, these tests are done only in some specialized centers and they are time consuming to perform. So, a rapid, reliable and *in vivo* test to distinguish between patients with and those without circulating functional autoantibodies would be of value to diagnose autoimmune urticaria and also to evaluate the effectiveness of immunomodulatory treatment. Intradermal injection of autologous serum in some patients can induce weal and flare response. This observation had led to the recognition of circulating autoantibodies in chronic urticaria and provides the basis of autologous serum skin test (ASST). Sensitivity and specificity of ASST are at best 80% respectively.[4]

## Indication

Suspected cases of autoimmune urticaria

## Prerequisites

1. Antihistamines should be withdrawn at least 2 to 3 days prior to the test.
2. Doxepin and Astemizole should be withdrawn 2 to 6 weeks beforehand.
3. The patient should not take immunosuppressants in the 2 months prior to the test.
4. Ethical approval should be taken from the appropriate body.
5. Age should be 18 years or more.
6. Test area should be free of lesion.

## Procedure

1. Two milliliters of venous blood is taken from antecubital vein.
2. Blood is allowed to undergo clotting at room temperature.
3. Serum is separated by centrifugation (2000 rpm for 10-15 min).
4. As much as 0.05 mL of serum is injected intradermally into the volar aspect of forearm, avoiding the areas of wealing within the past 24 h.
5. Equal amounts of normal saline [negative control] and histamine (10 μg/mL) [positive control] are injected intradermally 3 to 5 cm apart in the volar aspect of the same forearm.
6. Weal and flare responses are to be measured at 30 min. Redness of weal and flare reactions is difficult to perceive in pigmented skin types (e.g., Indian skin).[5] As erythema is contributed almost exclusively by histamine, omitting histamine control in patients of dark skin does not modify the result much. Rather for assessment of the ASST result, weal is much more relied upon.

## Criteria for Positivity

**Figure 1:**
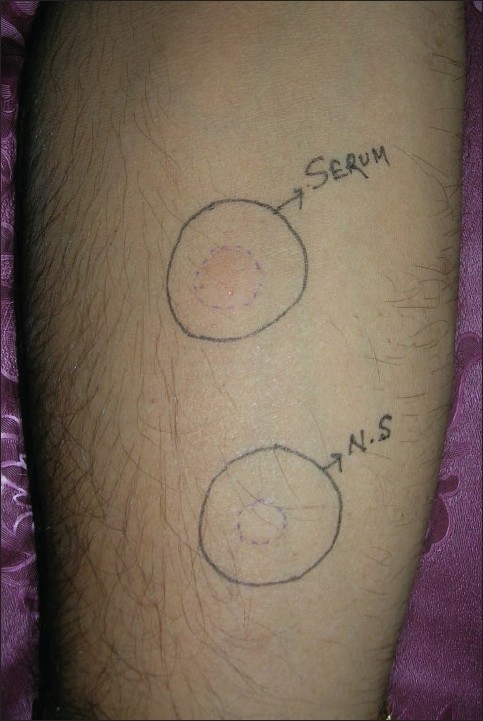
Positive autologous serum skin test. Serum-induced weal is larger than the normal saline (N.S.)–induced weal

A positive test is defined as a serum-induced weal response with a diameter of more than 1.5 mm or more than that of the saline-induced response at 30 min[6] [Figure 1]. First of all, the maximum vertical (d1) and horizontal (d2) diameters of the weals were measured. Then the average diameter (D) was calculated [D = (d1 + d2) / 2].

## Significance

Positive ASST denotes a subset of population that has an increased potential to develop urticaria due to endogenous causes as compared to patients without a positive test.[6] Moreover, a positive test has been found to correlate with the disease severity and period of the disease. Interestingly, positive test (ASST) has been reported to correlate strongly with patients who have multiple intolerances to nonsteroidal anti-inflammatory drugs. In some cases, positive ASST has been reported to be associated with the presence of *Helicobacter pylori* IgG antibodies. Patients with autoantibodies may need higher dose of antihistamine or additional immunomodulator. Recent findings indicate that ASST may be an indicator of the presence of circulating vasoactive factors rather than specific autoantibodies.[4] On the other hand, the significance of a negative test remains unclear.

## Causes of False Positive Results

1. Variations in injection technique, e.g., depth or volume of injections
2. Dermographic subjects

## References

[1] Hide M, Francis DM, Grattan CE, Hakimi J, Kochan JP, Greaves MW (1993). Autoantibodies against the high-affinity IgE receptor as a cause of histamine release in chronic urticaria. N Engl J Med.

[2] Fiebiger E, Hammerschmid F, Stingl G, Maurer D (1998). Anti-FcepsilonRIalpha autoantibodies in autoimmune-mediated disorders: Identification of a structure-function relationship. J Clin Investig.

[3] Niimi N, Francis DM, Kermani F, O'Donnell BF, Hide M, Kobza-Black A (1996). Dermal mast cell activation by autoantibodies against the high affinity IgE receptor in chronic urticaria. J Invest Dermatol.

[4] Sabroe RA, Greaves MW (2006). Chronic idiopathic urticaria with functional autoantibodies: 12 years on. Br J Dermatol.

[5] Sabroe RA, Grattan CE, Francis DM, Barr RM, Kobza Black A, Greaves MW (1999). The autologous serum skin test: A screening test for autoantibodies in chronic idiopathic urticaria. Br J Dermatol.

[6] Grattan C (2004). Autoimmune urticaria. Immunol Allergy Clin North Am.

